# Early detection of colorectal cancer based on presence of methylated syndecan-2 (*SDC2*) in stool DNA

**DOI:** 10.1186/s13148-019-0642-0

**Published:** 2019-03-15

**Authors:** Yoon Dae Han, Tae Jeong Oh, Tae-Ha Chung, Hui Won Jang, Youn Nam Kim, Sungwhan An, Nam Kyu Kim

**Affiliations:** 1 Division of Colorectal Surgery, Department of Surgery, Severance Hospital, Yonsei University College of Medicine, 50-1 Yonsei-ro Seodaemun-gu, Seoul, 03722 South Korea; 2 Genomictree, Inc., 44-6 Techno 10-ro Yuseong-gu, Daejeon, 34027 South Korea; 3 Department of Health Promotion, Severance check up, Health Promotion Center, Severance Hospital, 10 Tongil-ro, Jung-gu, Seoul, 04527 South Korea; 4 Division of Clinical Data Management Research, Clinical Trials Center, Severance Hospital, Yonsei University Health System, 50-1 Yonsei-ro Seodaemun-gu, Seoul, 03722 South Korea

**Keywords:** Colorectal cancer, DNA methylation, Biomarker, Early detection, Syndecan-2

## Abstract

**Background:**

Colorectal cancer (CRC) screening can effectively reduce disease-related mortality by detecting CRC at earlier stages. We have previously demonstrated that the presence of *SDC2* methylation in stool DNA is significantly associated with the occurrence of CRC regardless of clinical stage. The aim of this study was to evaluate the clinical performance of stool DNA-based *SDC2* methylation test for CRC.

**Methods:**

Aberrant *SDC2* methylation in stool-derived DNA was measured by linear target enrichment (LTE)-quantitative methylation-specific real-time PCR (qMSP). Duplicate reactions of me*SDC2* LTE-qMSP test were performed for stool samples obtained from CRC patients representing all stages (0–IV) and asymptomatic individuals who were subsequently underwent colonoscopy examination. To determine the diagnostic value of test in CRC and control groups, sensitivity and specificity were evaluated by receiver operating characteristic curve analysis.

**Results:**

Of 585 subjects who could be evaluated, 245 had CRC, 44 had various sizes of adenomatous polyps, and 245 had negative colonoscopy results. Stool DNA-based me*SDC2* LTE-qMSP showed an overall sensitivity of 90.2% with AUC of 0.902 in detecting CRC (0–IV) not associated with tumor stage, location, sex, or age (*P* > 0.05), with a specificity of 90.2%. Sensitivity for detecting early stages (0-II) was 89.1% (114/128). This test also detected 66.7% (2/3) and 24.4% (10/41) of advanced and non-advanced adenomas, respectively.

**Conclusions:**

Results of this study validated the capability of stool DNA based-*SDC2* methylation test by LTE-qMSP for early detection of CRC patient with high specificity.

**Trial registration:**

ClinicalTrials.gov, NCT03146520, Registered 10 May 2017, Retrospectively registered; however, control arm was prospectively registered.

## Background

Colorectal cancer (CRC), the third most common malignancy, is still one of the major causes of cancer-related mortality worldwide [1, 2]. Its incidence rate is rapidly increasing in many Asian countries including South Korea [3]. More surprisingly, the mortality of CRC ranked third in South Korea based on the 2016 Korean national cancer statistics [4], meaning that early detection rate was low. The mean 5-year survival rate for early-stage CRC can be as high as 90%. However, it can be less than 10% in patients with metastatic disease [5]. Therefore, early detection of CRC has emerged as an important global issue to reduce its high mortality. Many countries have population-based CRC screening programs [6]. Currently, colonoscopy is the most accurate screening method for early diagnosis of CRC. However, its compliance rate remains very low due to its invasiveness, dietary restriction requirement, and extensive bowel preparation [7, 8]. Although noninvasive fecal immunochemical tests (FIT) for hemoglobin in stool are available, their sensitivities are relatively low in detecting stage I CRC (53%) and advanced adenomas (≥ 1.0 cm) (27%), with a specificity of 94.6% [9]. Thus, developing highly accurate CRC screening method using molecular biomarkers for people who are reluctant to participate in colonoscopy examination is urgently needed for early detection of CRC.

Tumorigenesis of CRC arises through accumulation of genetic and epigenetic abnormalities of the genome [10]. Aberrant DNA methylation at CpG sites of genes is one of the most common molecular alterations during tumorigenesis of all types of cancer [11–13]. Aberrant methylation at specific CpG sites across the genome has been investigated, and in some instances was applied as biomarkers for early CRC detection.

Stool specimens from CRC patients usually contain exfoliated tumor cells. Detecting methylated DNA of a specific gene in noninvasive stool samples is a promising option to achieve detection of CRC [14]. Therefore, noninvasive stool-derived DNA testing has emerged as a new strategy for detecting of patients with both CRC and precancerous lesions. Stool DNA-based DNA methylation assays using several epigenetic biomarkers such as *BMP3*, *NDRG4*, *SDC2*, *SFRP2*, *TFPI2*, and *VIM* have been reported as potential noninvasive tools for early CRC detection [8, 15–21], with sensitivities ranging from 46 to 90% and specificities ranging from 76.8 to 93%. Cologuard (Exact Sciences), a multi-target stool DNA test that measures two methylation biomarkers (*BMP3*, *NDRG4*) and seven site mutations of *KRAS* in addition to FIT test in stool sample, has been approved by FDA in the USA [15].

Previously, we have identified aberrant *SDC2* methylation frequently occurring in all stages of CRC through comprehensive methylation analysis of CRC and normal mucosal tissue samples [22]. More recently, we have developed a highly sensitive and accurate methylation DNA assay consisting of quantitative methylation-specific real-time PCR (qMSP) following linear target enrichment (LTE) (named as me*SDC2* LTE-qMSP) for *SDC2* methylation and demonstrated that *SDC2* methylation test in stool DNA has high potential as a diagnostic method for early detection of CRC [16]. Several studies have also reported that *SDC2* methylation can be sensitively and specifically detected in stool and blood samples from CRC patients [8, 23–27].

In the present study, we described details of the analytical performance of me*SDC2* LTE-qMSP as a test for stool DNA samples for early CRC detection. The aim of this clinical trial was to determine the clinical performance of the stool DNA-based me*SDC2* LTE-qMSP test in detecting CRC in patients at various stages and precancerous lesions with various sized polyps.

## Methods

### Study design, population, and clinical procedures

**Fig. 1:**
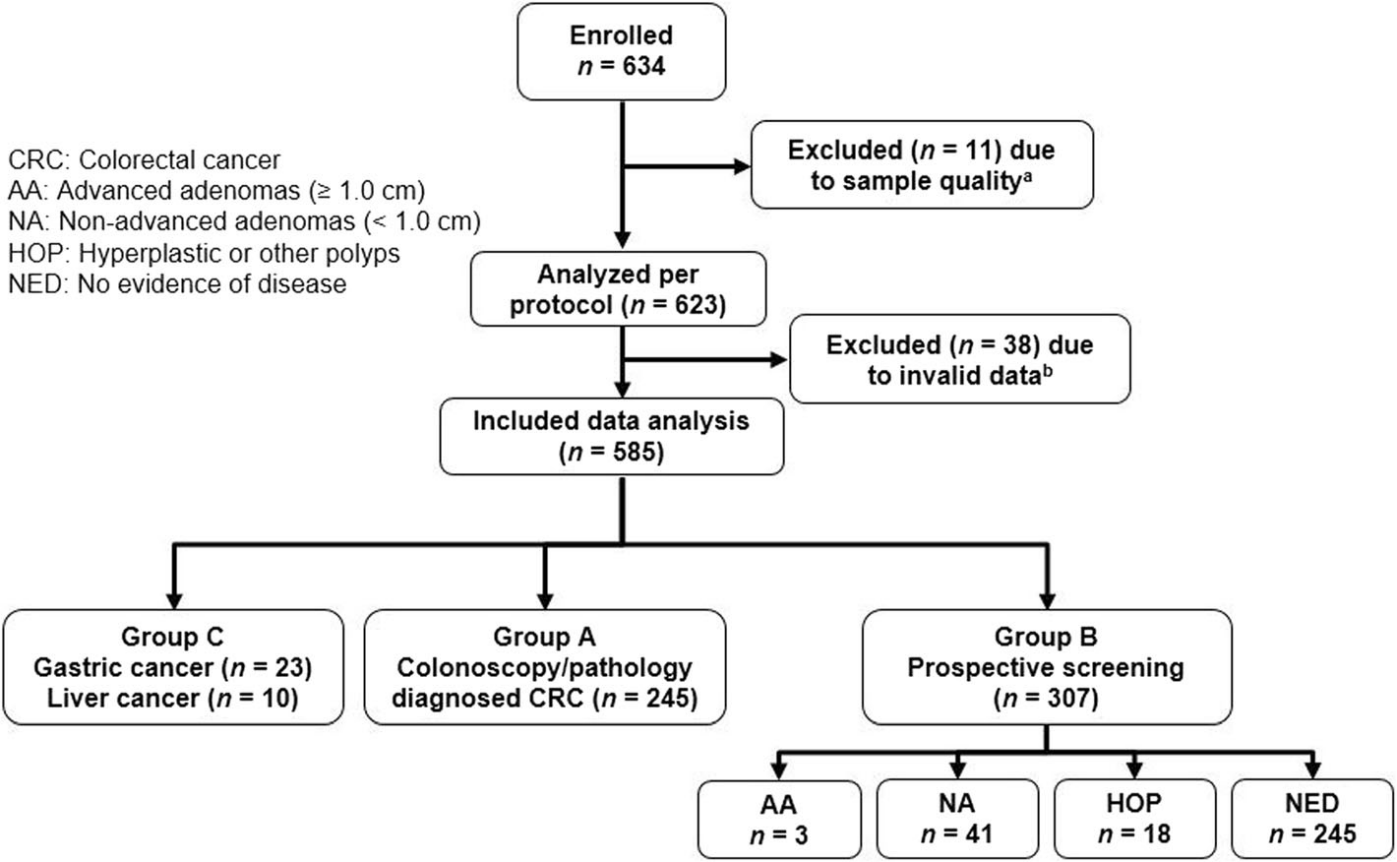
Study enrollment and flow diagram. The number of enrolled subjects according to diagnosis and the number of stool samples analyzed in this study. **a** Samples had insufficient DNA, **b** Samples either had insufficient human genomic DNA (low COL2A1) or unknown clinical stage

This retrospective case and prospective control study was designed to evaluate the clinical performance of stool DNA-based me*SDC2* LTE-qMSP test. All participants were enrolled from January 2017 to February 2018 at two clinical sites. Eligible subjects aged 30–80 years who provided written informed consent were enrolled. All prospective participants were recruited at Severance check up, Health Promotion Center, Severance Hospital (Seoul, South Korea). A single stool sample was collected at least 1 day before routine bowel preparation for undergoing screening colonoscopy. All colonoscopies were performed by board-certified endoscopists. Based on results of complete colonoscopy and pathology outcome, subjects were categorized as follows: advanced adenoma (AA, ≥ 1.0 cm), non-advanced adenoma (NA, < 1.0 cm), hyperplastic or other polyps (HOP), and no evidence of disease (NED, negative results on colonoscopy). Retrospective patients who had confirmed CRC and other gastrointestinal cancers were enrolled from Severance Hospital, Yonsei University Health System (Seoul, South Korea). A single stool sample was collected at least one day prior to curative surgery (Fig. 1).

me*SDC2* LTE-qMSP results were independently analyzed by comparison with colonoscopic findings and pathology outcomes as reference standards. No dietary or medication restrictions were required. Details of stool collection procedure are described in Supplemental Material. All statistical procedures were performed by the Clinical Trial Center at Severance Hospital. All personnel involved in experimental work were blinded to identities of samples.

We excluded participants who had previous history of CRC, any chemotherapy or radiotherapy, or incomplete information. The following information was collected: age, sex, clinical diagnosis, the number and size of polyps, histology subtype, and date of stool sample collection.

This clinical study was approved by the Institutional Review Board (IRB) of Severance Hospital, Yonsei University Health System (IRB No. 1–2016-0068). This clinical trial was sponsored and designed by Genomictree Inc. (Daejeon, South Korea) and registered at ClinicalTrials.gov (http://www.clinialtrials.gov, Trial Registration ID: NCT03146520). All authors had access to the study data. All authors have reviewed and approved the final manuscript.

### Stool collection, DNA isolation, and bisulfite treatment

Collection paper (JeongHyun MED, South Korea) was mounted to toilet seat to prevent contamination of toilet water. At least 2 g of stool was collected from four to five different spots into 20 ml of preservative buffer (Genomictree, Inc., South Korea) using a disposable spatula for each subject. All stool samples were shipped at ambient temperature to a central laboratory.

Stool DNA was isolated according to published protocol [16] with slight modifications. Briefly, all stool samples were weighed and thoroughly homogenized in preservative buffer using a multiple vortex mixer (MIULAB, China). After homogenization, 1~2 g equivalent of each stool sample was centrifuged at 2000 rpm for 2 min (HA-1000-3, Hanil Science Medical, Daejeon, South Korea). Supernatants were discarded and then 5.0 ml of GT buffer 1 (Genomictree, Inc. Daejeon, South Korea) was added to the pellet followed by incubation at room temperature for 4 min. Samples were then centrifuged at 2000 rpm for 2 min (HA-1000-3, Hanil Science Medical, Daejeon, South Korea) and supernatants were discarded. Subsequently, 2.0 ml of GT buffer 2 (Genomictree, Inc. Daejeon, South Korea) and 30 μl of proteinase K (0.4 mg/ml, Sigma-Aldrich, St. Louis, MO, USA) were added to each sample and incubated at 70 °C for 10 min. These samples were centrifuged at 3000 rpm for 5 min (HA-1000-3, Hanil Science Medical, Daejeon, South Korea). Then 0.75 ml of the supernatant was aliquoted to MaXtract High Density tube (Qiagen, Hilden, Germany) with 0.75 ml of Tris-saturated phenol-chloroform-isoamylalcohol (25:24:1 by volume) (Sigma-Aldrich, St. Louis, USA). Samples were thoroughly mixed for 1 min and centrifuged at maximum speed for 10 min in a microcentrifuge. Then 0.5 ml of the supernatant was transferred to a new tube containing 1/10 volume of 3 mol/L sodium acetate (pH 5.2, Welgene, Gyeongsan, South Korea) and equal volume of isopropanol (Merck, Darmstadt, Germany). Total nucleic acid was then precipitated by centrifugation at maximum speed for 10 min. DNA pellet was washed with 1.0 ml of 70% ethanol and dried. DNA concentration was measured using Qubit dsDNA BR assay kit (Thermo Fisher Scientific, MA, USA).

Stool-derived genomic DNA was chemically modified with sodium bisulfite using EZ DNA Methylation-Gold kit (ZYMO Research, USA) according to the manufacturer’s instructions. Bisulfite-converted DNA was either used immediately for methylation analysis or stored at -− 20 °C until further use.

### Analytical performance of me*SDC2* LTE-qMSP test

Analytical performance of me*SDC2* LTE-qMSP was determined based on real-time PCR using Rotor-Gene Q instrument (Qiagen, Germany). To determine the limit of detection (LoD) for methylated *SDC2* DNA, we prepared a mimic of natural sample matrix of clinical stool samples as follows. Different amounts (20, 2, 0.2, 0.1, 0.05, 0.02, 0.01, and 0 ng) of HCT116 genomic DNA as fully methylated human genomic DNA were diluted into 2.0 μg of *SDC2* methylation-negative stool genomic DNA pooled from 20 healthy individuals. Stool genomic DNA from these healthy individuals was confirmed as *SDC2* methylation-negative by LTE-qMSP. Resulting DNA samples from each concentration were pooled and divided to 24 aliquots so that the same DNA substrate was utilized for PCR. LoD, the point at which 95% of replicates of *SDC2* methylated DNA were detected, was determined by Probit analysis [28].

Reproducibility, repeatability, and lot-to-lot variation were evaluated according to Clinical Laboratory Standards Institute guideline EP05-A2 [29]. Tests were performed using *SDC2* methylated HCT116 genomic DNA with two concentrations (0.5 ng and 0.1 ng) diluted in 2 μg of *SDC2* methylation-negative stool genomic DNA pooled from 20 healthy subjects. *SDC2* methylation-negative stool DNA was also included. Reproducibility was assessed by testing duplicates of each concentration in two runs for 5 days at two different sites. Repeatability was evaluated by testing duplicates of each concentration in two runs for 20 days. The contribution of lot-to-lot variation was analyzed by testing duplicates of each concentration in two runs for 5 days. To evaluate the precision, we calculated mean *C*_T_, standard deviation (SD), and % of coefficient of variation (CV).

Assay specificity of the test kit was assessed by testing each 0.5 ng of HCT116 and 20 ng of *SDC2* methylation-negative genomic DNA spiked with 10^5^ to 10^6^ genome copies of DNA from the following bacteria and virus potentially found in humans: *Candida albicans*, *Staphylococcus epidermis*, *Streptococcus pneumoniae*, *Escherichia coli*, *Human Cytomegalovirus*, *Herpes simplex virus 1*, *Herpes simplex virus 2*, and *Hepatitis B virus*.

The effect of interfering substances on assay performance was evaluated using methylated *SDC2*-positive and negative stool samples spiked with 23 potential interfering substances selected based on clinical applications and diet habits in South Korea (Table 1).

**Table 1:** Potentially interfering substances tested in this study

No.	Interfering substances	Concentration per g stool
1	Kanamycin	2 mg
2	Streptomycin	2 mg
3	Trimethoprim	0.3 mg
4	Vancomycin	3.1 mg
5	Povidone iodine	10 mg
6	Paramoxine hydrochloride	19 mg
7	Berberine hydrochloride	9.23 mg
8	Dulcolax	10 mg
9	Glycerin	10 μl
10	Domperidone	0.14 mg
11	Omeprazole	2.83 mg
12	Cimetidine	2.06 mg
13	Vitamin U	6.84 mg
14	Cefixime	20.47 mg
15	Paracetamol	0.27 mg
16	Levofloxacin hydrochloride	0.15 mg
17	Ibuprofen	0.08 mg
18	Plant DNA	0.5 μg
19	Animal DNA	0.5 μg
20	Vegetable oil	20 μl
21	Bilirubin	450 μM
22	Ethanol	10 μl
23	Aspartame	0.1 mg

### me*SDC2* LTE-qMSP test in stool DNA and data analysis

Training of assay procedure and instruments for staff members were completed before the start of this study. me*SDC2* LTE-qMSP test was performed in duplicate reactions for each sample. In order to measure *SDC2* methylation, LTE was introduced to specifically enrich methylated *SDC2* target DNA from bisulfite modified DNA. Additionally, the region lacking CpG dinucleotides of *COL2A1* gene was used as a control to estimate the amount of amplifiable template and adequacy of bisulfite conversion. A total of 20 μl of reaction mixture containing 2.0 μg of bisulfite-converted stool DNA, 50 nmol/L each of *SDC2* methylation-specific antisense and *COL2A1* gene-specific antisense primers attached to 5′ universal sequence, and 4 μl of 5xAptaTaq PCR master mix (Roche Diagnostics, Swiss) was prepared. Thermal cycling conditions were as follows: 95 °C for 5 min followed by 35 cycles of 95 °C for 15 s and 60 °C for 60 s.

After LTE, the reaction mixture volume was scaled up to 40 μl, containing 8 μl of 5xAptaTaq PCR master mix, 250 nmol/L of *SDC2* methylation-specific sense primer, 125 nmol/L of *SDC2* probe, 125 nmol/L of *COL2A1* sense primer, 62.5 nmol/L of *COL2A1* probe, and 250 nmol/L of universal sequence primer. Thermal cycling conditions were as follows: 95 °C for 5 min followed by 40 cycles of 95 °C for 15 s and 60 °C for 60 s. Heating and cooling rates were 20 °C per second and 15 °C per second, respectively. For each run, bisulfite-converted methylated (HCT116) and unmethylated genomic DNA (whole genome amplified human lymphocyte DNA) were used as methylation controls. Non-template and non-template bisulfite-converted controls were also included.

PCR reactions for *SDC2* and *COL2A1* control were run in a single tube. me*SDC2* LTE-qMSP was performed on a Rotor-Gene Q real time PCR instrument (Qiagen, Germany). Cycle threshold (*C*_T_) value was calculated using Rotor Gene Q software.

For PCR analysis, *SDC2* methylation was detected if *C*_T_ was less than 40 cycles. It was not detected if *C*_T_ was not measurable [16]. Because stool samples contained very low levels of human DNA, *SDC2* methylation was called positive if at least one out of two PCR reactions had detectable methylated *SDC2* in order to maximize clinical sensitivity. Samples were categorized as positive if only one out of two reactions (1/2 algorithm) had detectable *SDC2*. Samples were considered negative if *SDC2* methylation was not measurable in the two reactions. Test result was acceptable only when *C*_T_ value of *COL2A1* was ≤ 31. If *COL2A1* was not detected or *C*_T_ value was more than 31, the test was re-run.

### Statistical analysis

Sample size was calculated using PASS 13.0 program (NCSS, USA). A sample size of 241 for CRC patients and 241 for subjects with NED would have a power of minimum 90% to detect each 10% higher at a one-sided type I error rate of 0.025 to test the following major hypotheses:( i) the sensitivity of *SDC2* methylation test is higher than 70%; and (ii) the specificity of *SDC2* methylation test is higher than 80%. Thus, samples were collected from 268 CRC patients and 268 subjects with NED in anticipation of a 10% of sample loss. We also additionally recruited patients with colorectal polyps (*n* = 67), gastric cancers (*n* = 23), and liver cancers (*n* = 12).

All statistical analyses of data were performed using SAS V9.4 (SAS Institute, NC, USA). To calculate sensitivity and specificity, test results were used in a dichotomous manner: methylation-positive as ‘1’ and methylation-negative as ‘0.’ Sensitivity and specificity of me*SDC2* LTE-qMSP for CRC detection were estimated as follows:

$$ \mathrm{Sensitivity}\ \left(\%\right)=\mathrm{true}\ \mathrm{positives}/\mathrm{a}\ \mathrm{total}\ \mathrm{number}\ \mathrm{of}\ \mathrm{CRC}\ \mathrm{patients}\times 100 $$

$$ \mathrm{Specificity}\ \left(\%\right)=\mathrm{true}\ \mathrm{negatives}/\mathrm{a}\ \mathrm{total}\ \mathrm{number}\ \mathrm{of}\ \mathrm{subjects}\ \mathrm{with}\ \mathrm{NED}\times 100 $$

The area under receiver operating characteristic (ROC) curve (AUC) and 95% confidence interval (CI) were calculated. Demographic and other clinical characteristics are described as frequency and percentile or mean and standard deviation. If *P* value was less than 0.05, the result was considered statistically significant.

## Results

### Analytical performance of me*SDC2* LTE-qMSP test

To determine the LoD for methylated *SDC2*, me*SDC2* LTE-qMSP tests were repeatedly (24 times) performed using samples with different concentrations of HCT116 genomic DNA. The test was able to detect methylated *SDC2* in stool DNA samples with concentrations as low as 10 pg (corresponding to ~ 3 diploid genome copies) in 54.2% (13/24) of replicates (Table 2).

**Table 2:** LoD of methylated *SDC2* DNA using HCT116 genomic DNA samples

DNA concentration (ng) diluted in 2.0 μg of stool genomic DNA	*SDC2* detected (%)	Average *C*_T_
20	24 out of 24 (100)	19.01
2.0	24 out of 24 (100)	22.43
0.2	24 out of 24 (100)	25.55
0.1	24 out of 24 (100)	26.92
0.05	23 out of 24 (95.8)	28.62
0.02	22 out of 24 (91.7)	30.08
0.01	13 out of 24 (54.2)	30.39
0	0 out of 24 (0)	Not detected

Number of positive test results out of the number of replicates tested and average *C*_T_ values for various concentrations of methylated SDC2

**Fig. 2:**
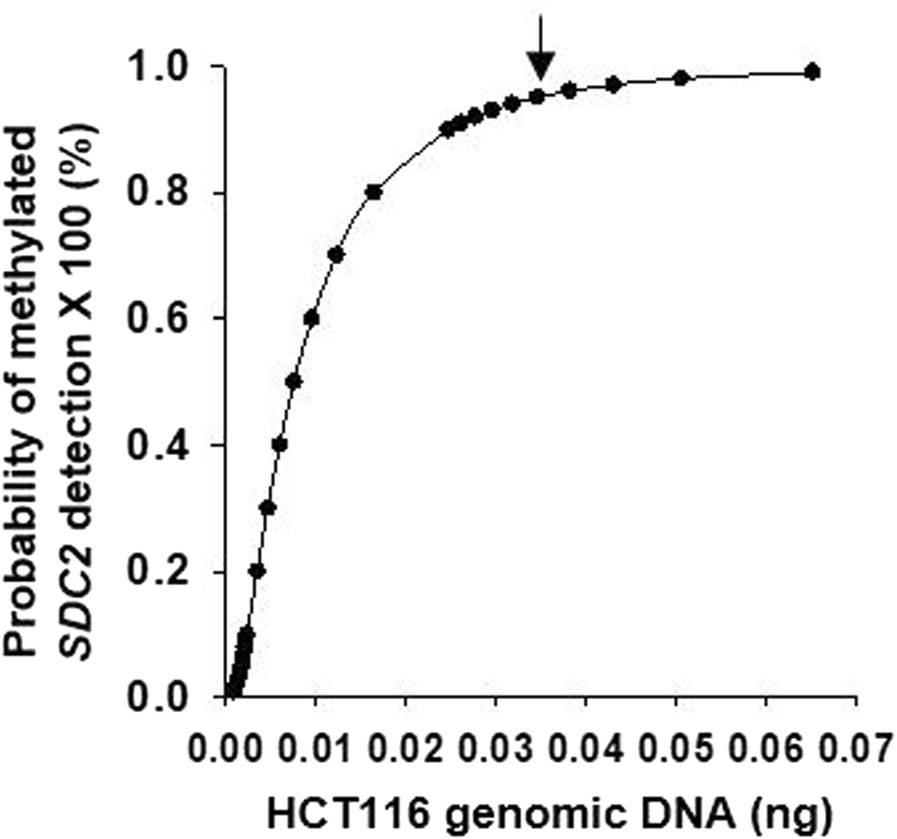
LoD of *SDC2* methylation test using data derived from Table 2. The LoD of the test using simulated stool DNA samples was determined by Probit analysis. The *y*-axis plots the probability of methylated SDC2 detection while the *x*-axis plots spiked HCT116 genomic DNA in a total of 2.0 μg of SDC2 methylation-negative stool genomic DNA. The estimated LoD (34.5 pg) is indicated by an arrow

The detection rate for 50 pg of methylated *SDC2* DNA was 95.8% (23/24). Probit analysis revealed that 95% LoD of me*SDC2* LTE-qMSP test kit was 34.5 pg (95% CI 18.6–64.1 pg) of methylated *SDC2* DNA, corresponding to ~ 20 diploid genome copies (Fig. 2).

Precision evaluation results of me*SDC2* LTE-qMSP test are described in Table 3. It was considered acceptable if CV was less than 5%. The test showed acceptable reproducibility, with CV ranging from 1.12 to 2.71%. Repeatability was also acceptable, with CV ranging from 2.13 to 2.68%. The lot-to-lot variation was low and acceptable, with CV ranging from 1.12 to 2.71%. Total precision achieved CV ≤ 5.0% for 20 days with concentrations of methylated *SDC2* DNA at 100 pg and 500 pg. For 2.0 μg of *SDC2* methylation-negative stool genomic DNA (40 samples), negative agreement was 100%.

**Table 3:** Precision evaluation results of the me*SDC2* LTE-qMSP test kit

Target	HCT116 genomic DNA concentration (pg)	Mean *C*_T_ (%CV)				
		Reproducibility		Repeatability	Lot-to-lot variation	
		Site 1	Site 2		Lot 1	Lot 2
*SDC2*	100	26.19 (2.71)	25.75 (2.10)	26.1 (2.68)	26.19 (2.71)	25.75 (2.10)
	500	23.52 (2.21)	23.15 (1.12)	23.44 (2.13)	23.52 (2.21)	23.15 (1.12)

Interfering substances tested in this study had no effect on test performance of me*SDC2* LTE-qMSP. The test kit showed no cross-reactivity even when excess amounts (10^5^–10^6^ genome copies) of bacterial and viral DNA were tested.

### Study population

This clinical trial enrolled a total of 634 participants who visited the hospital for routine colonoscopies or had confirmed CRC or other gastrointestinal cancer diagnosis at two clinical sites. A total of 585 (92.3%) of 634 participants had results that could be fully evaluated.

Of 245 subjects enrolled in group A, 3, 55, 70, 96, and 21 subjects were at stages 0, I, II, III, and IV of CRC, respectively. In group B patients, 41, 3, and 18 subjects were found to have NAs, AAs, and HOP, respectively. The remaining study participants were subjects with NED (*n* = 245). Group C had confirmed diagnosis of gastric cancer (*n* = 23) or liver cancer (*n* = 10). Gastric or liver cancer patients did not undergo colonoscopy examination. Demographic data for subjects fully evaluated in this study are shown in Table 4.

**Table 4:** Demographic features of subjects evaluated in this study

Demographic					Clinical diagnosis of prospectively enrolled patients			
		CRC^b^	Gastric cancer	Liver cancer	AA^a^	NA^a^	Hyperplastic/other polyp	NED^b^
		*n* (%)	*n* (%)	*n* (%)	*n* (%)	*n* (%)	*n* (%)	*n* (%)
Sex	Male	133 (54.3)	9 (39.1)	8 (80.0)	2 (66.7)	22 (53.7)	12 (66.7)	114 (46.5)
	Female	112 (45.7)	14 (60.9)	2 (20.0)	1 (33.3)	19 (46.3)	6 (33.3)	131 (53.5)
Age	≤ 49	36 (14.7)	9 (39.1)	1 (10.0)	–	11 (26.8)	4 (22.2)	87 (35.5)
	50–59	62 (25.3)	7 (30.4)	5 (50.0)	1 (33.3)	17 (41.5)	9 (50.0)	105 (42.9)
	60–69	79 (32.2)	5 (21.7)	3 (30.0)	2 (66.7)	12 (29.3)	5 (27.8)	47 (19.2)
	≥ 70	68 (27.8)	2 (8.7)	1 (10.0)	–	1 (2.4)	–	6 (2.4)
Total		245	23	10	3	41	18	245

^a^*AA* advanced adenomas (≥ 1.0 cm), *NA* non-advanced adenomas (< 1.0 cm)

^b^*CRC* colorectal cancer, *NED* no evidence of disease

### Clinical performance of me*SDC2* LTE-qMSP test for detecting CRC in stool-derived DNA

**Fig. 3:**
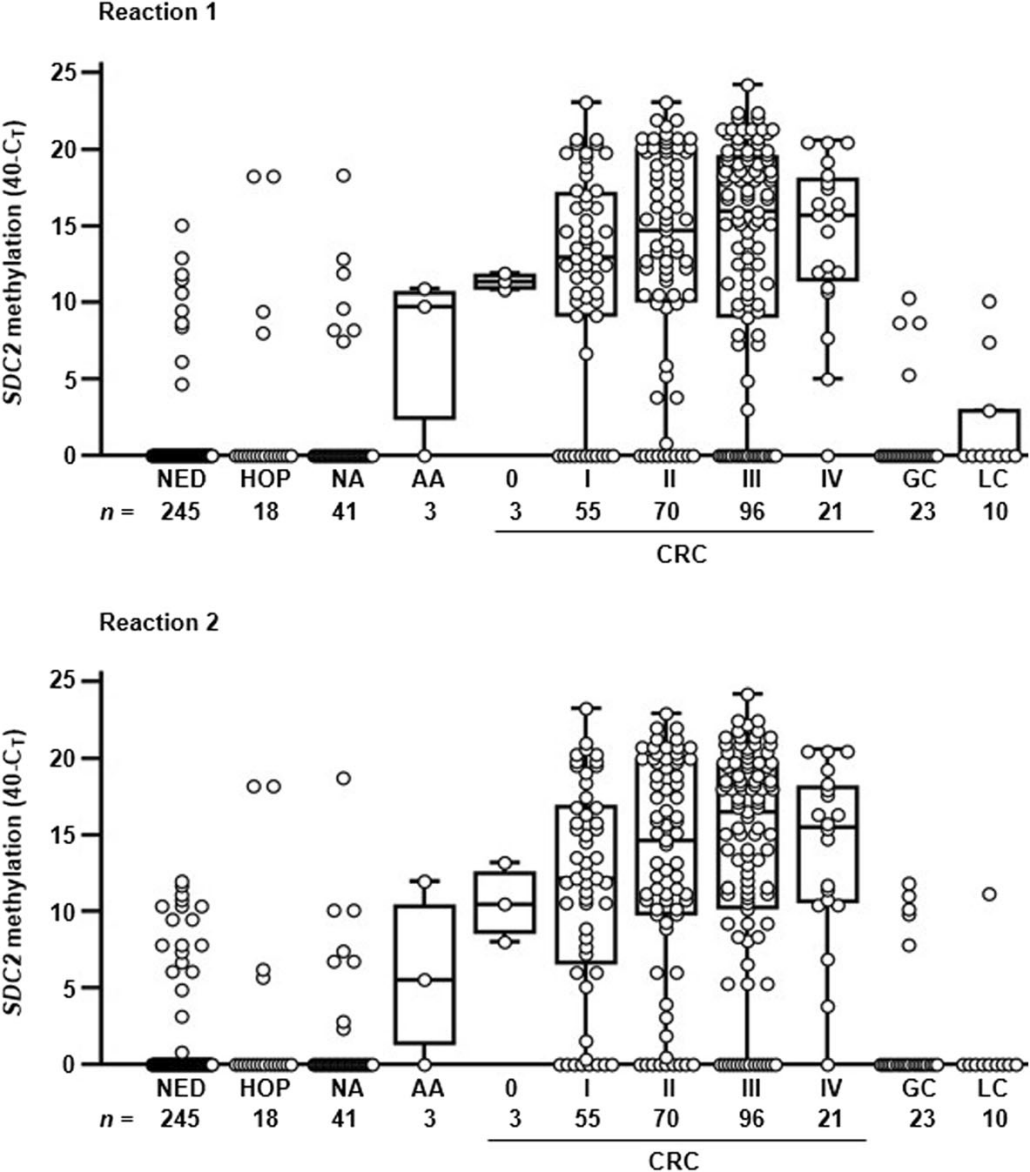
Results of SDC2 methylation analysis from two reactions in stool DNA. Distribution of SDC2 methylation was expressed in *C*_T_ values as 40-*C*_T_ for each sample. A higher 40-*C*_T_ represents a higher methylation level of SDC2. It is represented as 0 if the SDC2 methylation was not detectable. Methylation status of SDC2 gene is plotted as box and whisker plots. *CRC* colorectal cancer (stage 0–IV), *HOP* hyperplastic or other polyps, *NA* non-advanced adenomas (< 1.0 cm), *AA* advanced adenomas (≥ 1.0 cm), *GC* gastric cancer, *LC* liver cancer, *NED* no evidence of disease

To evaluate the clinical performance of me*SDC2* LTE-qMSP test for detecting CRC using stool DNA, we tested 585 valid samples, including 245 CRCs (0-IV), 3 AAs (≥ 1.0 cm), 41 NAs (< 1.0 cm), 18 HOP, 33 other gastrointestinal cancers, and 245 subjects with NED*.* Of 245 CRC samples, 128 (52.2%) were obtained from patients with early stages (0–II) of CRC. me*SDC2* LTE-qMSP test showed higher frequency and higher level of aberrant *SDC2* methylation in both CRC and adenomas patients than that in subjects with NED (Fig. 3).

**Fig. 4:**
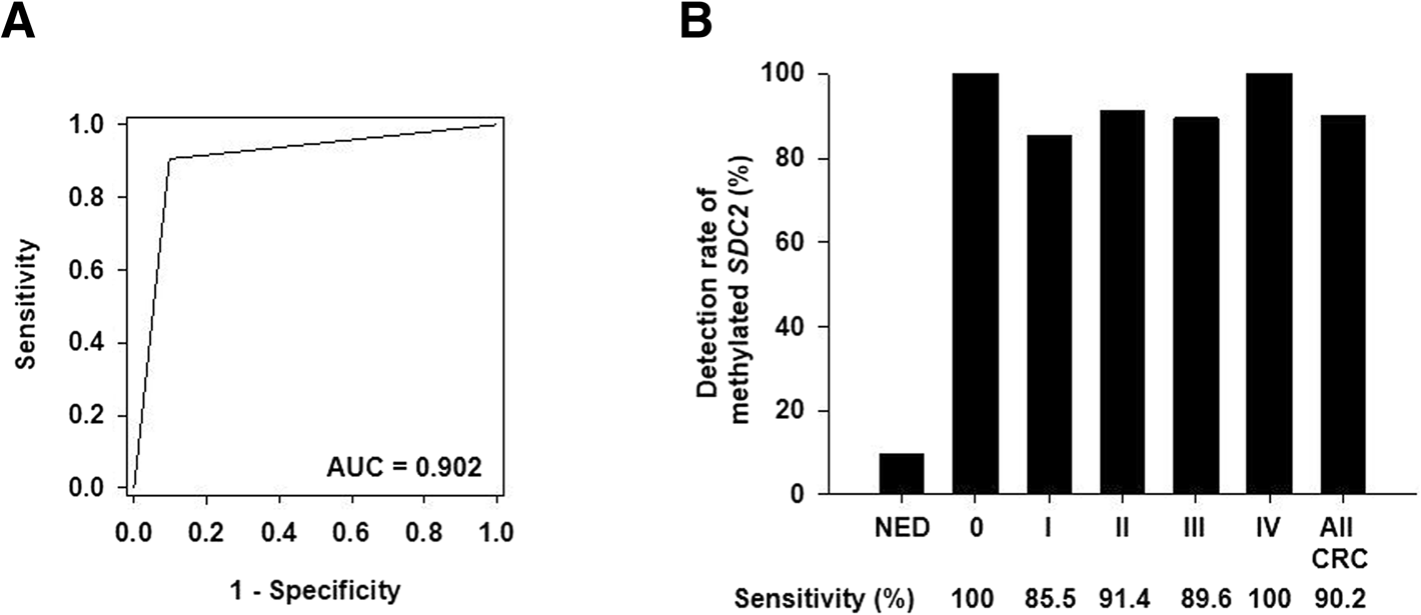
Results of stool DNA-based SDC2 methylation test. **a** ROC curve was plotted for CRC patients vs. subjects with NED. AUC is indicated. **b** Sensitivity of SDC2 methylation test for detecting CRC according to clinical stages. Percent of samples with detectable methylated SDC2 DNA using 1/2 algorithm is depicted by solid bars

To evaluate the clinical performance of *SDC2* methylation test in stool DNA to detect CRC (0–IV), ROC curve was constructed using pre-specified cutoff value [16] of *C*_T_ 40 on test results (Fig. 4a). me*SDC2* LTE-qMSP test in stool DNA had an overall sensitivity of 90.2% (221/245, 95% CI 85.8–93.6%) with AUC of 0.902 (95% CI 0.876–0.928). Sensitivities for individual stages 0, I, II, III, and IV were 100% (3/3), 85.5% (47/55), 91.4% (64/70), 89.6% (86/96), and 100% (21/21), respectively (Fig. 4b). For early stages (0–II) of CRC, sensitivity was 89.1% (114/128, 95% CI 82.3–93.9%). Sensitivity did not vary significantly (*P* > 0.05) according to cancer stage, tumor location, sex, or age. Detection rates of aberrant *SDC2* methylation in stool DNA samples from patients are summarized in Table 5. me*SDC2* LTE-qMSP test detected 66.7% (2/3) and 24.4% (10/41) of AAs and NAs, respectively. For HOP (< 1.0 cm), detection rate of *SDC2* methylation was 26.3% (5/19), similar to the detection rate for NAs. For gastric and liver cancers, detection rates of *SDC2* methylation were 30.4% (7/23) and 30 (3/10), respectively (Table 4). For 245 subjects with totally negative results on colonoscopy, the specificity was 90.2% (221/245, 95% CI 85.8–93.6%) (Fig. 4b). Within this group, specificities were 90.1% (173/192) and 90.6% (48/53) for subjects aged < 60 years and ≥ 60 years, respectively (*P* = 0.872, Chi-square test). Specificities for women and men were 90.4% (103/114) and 90.1% (118/131), respectively (*P* = 0.943, Chi-square test).

**Table 5:** Detection rate (%) of *SDC2* methylation in stool DNA from patients

Diagnosis	No. of samples	No. of *SDC2* methylation detected (%)
Adenomas	44	12 (27.3)
NA (< 1.0 cm)	41	10 (24.4)
AA (≥ 1.0 cm)	3	2 (66.7)
*P* value^a^	0.360	
CRC	245	221 (90.2)
Sex		
Male	133	119 (89.5)
Female	112	102 (91.1)
*P* value^a^	0.675	
Age		
≤ 49	36	33 (91.7)
50–59	62	54 (87.1)
60–69	79	74 (93.7)
≥ 70	68	60 (88.2)
*P* value^a^	0.544	
Clinical stage		
0	3	3 (100)
I	55	47 (85.5)
II	70	64 (91.4)
III	96	86 (89.6)
IV	21	21 (100)
*P* value^b^	0.413	
Tumor location		
Ascending	34	28 (82.4)
Transverse	11	8 (72.7)
Descending	6	6 (100)
Sigmoid	91	85 (93.4)
Rectum	90	83 (92.2)
Others	13	11 (84.6)
*P* value^b^	0.108	
Gastric cancer	23	7 (30.4)
Liver cancer	10	3 (30.0)

^a^*P* value was calculated by Chi-square test

^b^*P* values were calculated by Fisher’s exact test

## Discussion

CRC screening is crucial because it is highly curable if the disease is detected at an early stage [5]. We have developed a highly sensitive and accurate stool DNA-based *SDC2* methylation biomarker test named me*SDC2* LTE-qMSP for early detection of CRC [16]. In this clinical trial, we evaluated the clinical performance of me*SDC2* LTE-qMSP test for detecting CRC in stool DNA. Results of this study demonstrated that the test had high sensitivity and specificity for early detection of CRC. Thus, stool DNA-based me*SDC2* LTE-qMSP test could be used as an alternative screening option for early CRC detection. It could be provided to individuals with average risk for CRC who are reluctant to undergo colonoscopy.

Conventional screening can effectively reduce death rate from CRC [30]. Nevertheless, only about 50% and 30% of the eligible population had participated in CRC screening programs in the USA and South Korea, respectively [31, 32]. Although colonoscopy and fecal occult blood test (FOBT) or FIT are the most widely used screening tools for CRC, these methods are limited by uptake and adherence [33–35]. Thus, the need for a noninvasive CRC screening tool using improved biomarkers has arisen. Early CRC detection will contribute to better treatment outcomes.

Colorectal tumor cells are constantly exfoliated into the lumen. Exfoliation of these tumor cells into stool logically occurs earlier than vascular invasion into blood during the progression of CRC development [36]. Thus, measurement of aberrant DNA methylation in stool samples is ideal for cancer-specific early detection of CRC [14]. Recently, stool DNA-based biomarker tests have provided attractive options for noninvasive screening of CRC [14–20, 37]. Meanwhile, stool matrices contain exfoliated epithelial cells, diets, bacteria, and various PCR inhibitors [38–40]. Thus, stool specimens are difficult for PCR amplification. We have previously developed a highly sensitive and accurate method called me*SDC2* LTE-qMSP test for methylation analysis of *SDC2* in stool DNA and demonstrated that stool DNA-based me*SDC2* LTE-qMSP test has a high potential for early CRC detection [16].

In the present study, we first provided details of analytical performance for me*SDC2* LTE-qMSP test. We then performed a clinical trial to evaluate the performance of the test using stool DNA. Analytical studies revealed that the test kit was able to detect methylated *SDC2* at single-digit copy number level. This provides strong evidence that me*SDC2* LTE-qMSP test is optimized for using stool-derived DNA.

Because *C*_T_ values for 90.9% of all subjects with NED were undetectable in the real-time PCR of our pilot study [16], we determined the sensitivity and specificity using a pre-specified cutoff (*C*_T_ 40) for *SDC2* methylation based on the presence or absence of detectable *SDC2* methylation without quantitating methylation in this clinical trial. To determine methylation status of *SDC2* in stool DNA, we performed PCR in two reactions to achieve the highest clinical sensitivity. If at least one out of two PCR reactions from a subject was positive, the test was considered as positive (1/2 algorithm). Overall sensitivity of me*SDC2* LTE-qMSP test for CRC (0–IV) was 90.2% with a specificity of 90.2%. These observed clinical sensitivity and specificity results were consistent with results of our previous study [16]. There was no significant difference in sensitivity between early and late stages of CRC (*P* = 0.679, Chi-square test). me*SDC2* LTE-qMSP test revealed that *SDC2* methylation-positive began to appear in patients with precancerous lesions. It occurred very frequently (over 90%) in CRC patients regardless of stage. However, it occurred in 10% of subjects with NED. The results indicate that the test is very useful for early detection of CRC. Meanwhile, Niu et al. [8] have recently published results of *SDC2* methylation test for stool DNA from Chinese population. Its overall accuracy for detecting CRC was comparable to ours. Assuming CRC prevalence of 0.5% in an average-risk population aged 50 years or older [41], stool DNA-based me*SDC2* LTE-qMSP test has a low positive predictive value (PPV) of 4.6% and a high negative predictive value (NPV) of 99.9%.

In our preliminary study, *SDC2* methylation-positive was very frequently detected about 85% in tissues of gastric cancer, whereas it was not detected in other solid cancers (data not shown). Aberrant *SDC2* methylation was also associated with Lauren classification subtype in early gastric tumorigenesis [42]. Therefore, we included gastric cancer patients to determine whether *SDC2* methylation was detectable in stool samples from gastric patients. For gastric cancer patients, detection rate of *SDC2* methylation was as low as 30.4%. This low detection rate of methylated *SDC2* in stool DNA implies that DNA from cells exfoliated by gastric mucosa is not easily detected in stool specimen. To determine CRC-specific methylation of *SDC2*, we also enrolled liver cancer patients. For liver cancer patients, detection rate of methylated *SDC2* was as low as 30%. This low percentage of *SDC2* methylation-positive suggests its low false positivity for liver cancer. On the other hand, considering that not all gastric and liver cancer patients were verified with colonoscopy as colorectal neoplasm-free, *SDC2* methylation-positive in those patients might indicate that they have colorectal neoplasm. To address this, colonoscopy-verified gastric and liver cancer patients need to be tested in the future.

Imperiale et al. [15] have reported that multi-target stool DNA test (Cologuard) has sensitivity of 92% for detecting CRC with PPV of 3.7%. Its specificity for subjects with negative results on colonoscopy was 90% with NPV of 99.9%. For subjects with NAs, non-neoplastic findings, and negative results on colonoscopy, the specificity was 87%. These results are comparable to our test results. However, multi-target stool DNA test is relatively expensive. It requires complicated analytical procedures due to the use of multiple markers and whole stool collection. Compared to multi-target stool DNA test, our stool DNA-based me*SDC2* LTE-qMSP test has several features including the use of single methylation biomarker, availability of transportation of stool samples in preservative buffer at ambient temperature, relative simplicity, and the use of partial stool collection.

To effectively reduce CRC mortality, the test must have high sensitivity because the primary aim of screening test is to detect cancer [43]. Because *SDC2* methylation was not detectable in most subjects with NED, combination of *SDC2* gene with *BMP3*, *NDRG4*, or other genetic and epigenetic biomarkers may improve its sensitivity for detecting CRC without losing specificity.

Our stool DNA-based me*SDC2* LTE-qMSP test had the same high sensitivity for detecting CRC as Cologuard. In terms of false positive rate, NPV of our test was comparable to that of Cologuard (99.9%) and FIT (99.7%), indicating that a negative methylation test result can provide similar information on the absence of CRC.

Meanwhile, MethHC database revealed that *SDC2* was frequently methylated in tissues from colorectal and gastric cancers, but less frequently or unmethylated in most of the other solid tumors or normal tissues from patients of European continental origin [44]. Thus, *SDC2* methylation does not seem to have ethnic difference.

This study has several limitations. The number of samples representing precancerous lesions of CRC was insufficient to evaluate its diagnostic value. Thus, more patients in this group need to be tested in future studies. Further studies are also needed to perform me*SDC2* LTE-qMSP test using stool DNA samples from patients with inflammatory bowel disease (IBD) to determine the potential influence of exfoliation of methylated *SDC2* DNA from small neoplasms in IBD on test results. The present study had retrospective cases with prospective control composite design. Therefore, multi-center prospective studies are needed for intensive evaluation of stool DNA-based me*SDC2* LTE-qMSP test in screening setting. Furthermore, testing intervals and cost-effectiveness of stool DNA-based *SDC2* methylation test in CRC screening setting should be further considered in the future.

In conclusion, this study demonstrated that stool DNA-based me*SDC2* LTE-qMSP test had a high diagnostic value for early detection of CRC. Our results imply that *SDC2* methylation test is a new potential diagnostic test for CRC using stool samples noninvasively. Further prospective cohort studies will be needed to determine the clinical utility of this test for population-based CRC screening.

## Abbreviations

AA: Advanced adenoma
AUC: Area under ROC curve
CI: Confidence interval
CRC: Colorectal cancer
FIT: Fecal immunochemical test
FOBT: Fecal occult blood test
HOP: Hyperplastic or other polyps
LTE-qMSP: Linear target enrichment-quantitative methylation-specific real-time PCR
NA: Non-advanced adenoma
NED: No evidence of disease
NPV: Negative predictive value
PPV: Positive predictive value
ROC: Receiver operating characteristics: *SDC2*: Syndecan-2
